# Trends in Weather Conditions and Performance by Age Groups Over the History of the Berlin Marathon

**DOI:** 10.3389/fphys.2021.654544

**Published:** 2021-05-13

**Authors:** Beat Knechtle, David Valero, Elias Villiger, José R. Alvero-Cruz, Pantelis T. Nikolaidis, Ivan Cuk, Thomas Rosemann, Volker Scheer

**Affiliations:** ^1^Medbase St. Gallen Am Vadianplatz, St. Gallen, Switzerland; ^2^Institute of Primary Care, University Hospital Zurich, Zurich, Switzerland; ^3^Ultra Sports Science Foundation, Pierre-Bénite, France; ^4^Departamento de Fisiología Humana, Histología, Anatomia Patológica y Educación Física y Deportiva, University of Málaga, Málaga, Spain; ^5^School of Health and Caring Sciences, University of West Attica, Athens, Greece; ^6^Laboratory of Exercise Testing, Hellenic Air Force Academy, Acharnes, Greece; ^7^Faculty of Physical Education and Sports Management, Singidunum University, Belgrade, Serbia

**Keywords:** running, heat, cold, rain, performance

## Abstract

The effect of different environmental conditions such as temperature, wind, barometric pressure, and precipitation has been well investigated in elite marathoners, but not by age categories (i.e., age group marathoners). The aim of the study was to investigate the potential influence of environmental conditions such as temperature, precipitation, and atmospheric pressure on marathon performance in age group marathoners competing in the ‘Berlin Marathon’ from 1974 to 2019. A total of 869,474 valid finisher records were available for analysis, of which 711,136 correspond to males and 158,338 to females. The influence of temperature, atmospheric pressure, and precipitation on marathon race times was investigated in age group marathoners grouped in 5-year-intervals. Within the 46 years of Berlin marathons under investigation, there was some level of precipitation for 18 years, and 28 years without any rain. Sunshine was predominant in 25 of the events, whilst in the other 21 years, cloud cover was predominant. Marathon race times were significantly and positively correlated with age (i.e., older runners were slower than younger runners) where the correlation was higher for males than for females. Marathon race times were significantly and positively correlated with both the hours of sunshine and the daily maximum temperature. The fastest marathon runners (meaning the minimum times) achieved the fastest race times on race days with higher maximum temperatures (i.e., 15–30°C). Daily maximum temperatures showed an influence on age group marathoners from age group 35–40 years and older. Higher precipitation levels impaired performance across most age groups. In summary, higher daily maximum temperatures (i.e., >15°C) and higher precipitation levels impaired performance of master marathoners (i.e., 35–40 years and older) competing in the ‘Berlin Marathon’ in the last 45 years. Master marathoners should start in marathon races with temperatures < 15°C and no precipitation in order to achieve a fast marathon race time.

## Introduction

Marathon running is of high popularity with a documented increase in participants in recent decades mainly in large city marathons such as the ‘New York City Marathon’ (Jokl et al., 2004; Vitti et al., 2020). The increase in marathoners in large city marathons is mainly due to an increase in both female (Vitti et al., 2020) and age group marathoners (i.e., master marathoners older than 35 years) (Jokl et al., 2004) and in particular of female master marathoners who increased their participation to a greater extent compared to male master marathoners (Lepers and Cattagni, 2012). In addition to the increase in participation, master marathoners of higher ages (i.e., 75 years and older) competed in these races and they also improved their performances in recent decades (Ahmadyar et al., 2015, 2016).

Regarding marathon performance, it is well-known that environmental conditions such as ambient temperature (Cheuvront and Haymes, 2001; Nikolaidis et al., 2019), wind (El Helou et al., 2012), cloud cover (Trapasso and Cooper, 1989; Ely et al., 2007a), barometric pressure (Knechtle et al., 2019; Nikolaidis et al., 2019), and precipitation (Trapasso and Cooper, 1989; Knechtle et al., 2019; Nikolaidis et al., 2019) have a considerable effect on marathon running performance. An analysis investigating marathon races times of the World Marathon Major races for Boston Marathon, London Marathon, Berlin Marathon, Chicago Marathon, and New York City Marathon showed that weather rather than course had an effect on marathon race times (Maffetone et al., 2017).

Among all the weather variables, ambient temperature seemed to have the highest influence on marathon race times (Zhang et al., 1992; Ely et al., 2007b). There is evidence that performance in a marathon race is impaired with increasing temperature (Trapasso and Cooper, 1989; Ely et al., 2007b; González-Alonso, 2007; El Helou et al., 2012). The optimum temperature for a fast marathon race time is generally at ∼10–12°C (Ely et al., 2007a; Maughan, 2010) or even lower at ∼8°C (Trapasso and Cooper, 1989).

The influence of temperature on marathon performance seemed, however, to depend on the performance level of a runner (Ely et al., 2008; El Helou et al., 2012) where the optimum temperature for a fast marathon race time may be lower for faster runners than for slower runners (Maughan, 2010). In some investigations, higher temperatures seemed to slow down faster runners compared to slower runners (Ely et al., 2008) whereas in other circumstances, slower runners suffered a greater performance decline in higher temperatures than faster runners (Montain et al., 2007; Vihma, 2010).

Based on these findings, we have knowledge that environmental conditions such as temperature, barometric pressure, cloud cover, and rain have a remarkable influence on marathon running performance regarding performance level (i.e., slower and faster runners), but we have no knowledge about the influence of environmental conditions on performance in age group marathoners (i.e., master marathoners). What we know from scientific literature is the fact that middle-aged and older adults have an impairment in performance in the heat (Larose et al., 2013; Stapleton et al., 2015; McGinn et al., 2017; Balmain et al., 2018) which is most probably due to a deterioration of the thermoregulatory response with advancing age (Bongers et al., 2014).

The aim of the present study was, therefore, to investigate the influence of different environmental conditions such as temperature (i.e., mean temperature and daily highest temperature on race day), sunshine duration, precipitation, barometric pressure on marathon race times in age group marathoners (i.e., master marathoners) competing in all editions of the ‘Berlin Marathon’ since its first edition in 1974 until 2019. Based upon the existing knowledge of the influence of heat on athletic performance in middle-aged and older adults, we assumed that higher ambient temperatures would impair marathon performance in master marathoners. However, also other variables such as sunshine duration, cloud cover, barometric pressure, and precipitation might have a minor influence on master marathoners.

## Materials and Methods

### Ethical Approval

This study was approved by the Institutional Review Board of Kanton St. Gallen, Switzerland, with a waiver of the requirement for informed consent of the participants as the study involved the analysis of publicly available data.

### Data Set and Data Preparation

The ‘Berlin Marathon’ was chosen due to the fact that ‘Berlin Marathon’ is the fastest marathon race course in the world^1^ and weather data from all editions since the first edition in 1974 was available. The athlete data with name, surname, year of birth, sex, and nationality was obtained directly from the website of the ‘Berlin Marathon’^2^. We were able to download the entire dataset for each year in JSON format and then convert it to an Excel file using a custom Python script (Python 2016, Python Software Foundation, United States). The weather data on the race day was downloaded from the website of ‘Deutscher Wetterdienst’^3^ with temperature (maximum, average in °C), sunshine (duration in hours), precipitation (mm), cloud cover (duration in hours), and atmospheric pressure (mbar) and filtered by the respective race dates. We chose the data from the weather station Berlin Dahlem because of its proximity to the ‘Berlin Marathon’ route.

### Data Processing

Two data files have been used in this study: A register of Berlin marathon runner’s finishing times between 1974 and 2019 (with the exception of 1978 and 1980 for which no data was available), including the runner’s finish time in the format HH:MM:SS, along with their sex and age, and the year of the marathon. The following age groups were defined: 18 (less than 20 years of age), 20 (20–29 years), 30 (30–34 years), 35 (35–39 years), 40 (40–44 years), 45 (45–49 years), 50 (50–54 years), 55 (55–59 years), 60 (60–64 years), 65 (65–69 years), 70 (70–74 years), 75 (75–79 years), and 80 (80 years of age or older). For each gender, race times in each age group were further filtered by each of the four originally continuous weather variables under consideration, converted into categories (ranges), as follows: Temperatures (°Celsius) grouped in three ranges: 0–8, 8–15, 15–30°C, atmospheric pressure values (mbar) in two ranges: 900–1013, 1013–1030 mbar, and precipitation values (mm) in three ranges: 0–10, 10–20, 20–50 mm. These groups are selected based on existing results from the Boston Marathon (Knechtle et al., 2019; Nikolaidis et al., 2019). A second register of the weather conditions on each marathon day between 1974 and 2019, including temperature values (average and maximum), and average atmospheric pressure and precipitation, along with sunshine and cloud cover hours. Sunshine is highly correlated with temperature, and cloud cover with sunshine, so only temperature is used in the analysis. These files were visually inspected on an Excel spreadsheet first, where minor changes were made (renaming of header columns and removing of unused columns) and then uploaded into a Google Colab^4^ notebook, where Python (Python Software Foundation^5^) was used to conduct the statistical processing and to create the results tables and charts. Given the main goal of performing descriptive statistics on the available data, the decision was made not to establish cut-off finish times on either end of the range.

### Statistical Analysis

Descriptive statistical analysis has been performed by age group, gender, and environmental conditions (multi-variable analysis). The resulting values of the marathon race times are presented in terms of their average value (mean) and standard deviation (std), along with maximum (max) and minimum (min) values for each category. The column named as “n” represents the number of samples in each specific category. The Kolmogorov–Smirnov two-sample test was applied to the male/female sub-populations to validate the assumption of the statistical significance of the resulting finishing times by gender. ANOVA two-way tests were run to explore the statistical significance of the differences observed in the finish times by age group and weather conditions. Statistical significance was set at 5% (*p* < 0.05) in all cases. All analyses were carried out using the Python programming language (Python Software Foundation, see footnote 5), on a Google Colab notebook (see footnote 4), and the Statistical Software for the Social Sciences (IBM SPSS v26. Chicago, Ill, United States).

## Results

After cleaning up and processing the data, a total of *n* = 869,474 valid finisher records were available for the analysis, of which 711,136 correspond to males and 158,338 to females.

**FIGURE 1:**
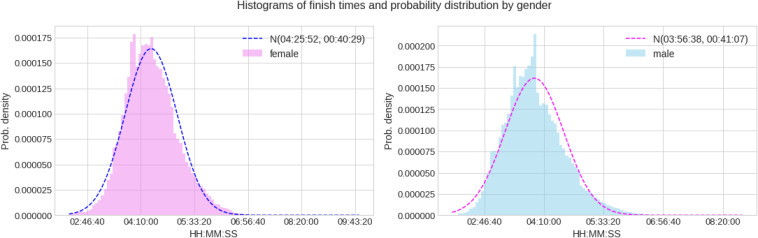
Marathon race time histograms and probability distributions for women and men.

**FIGURE 2:**
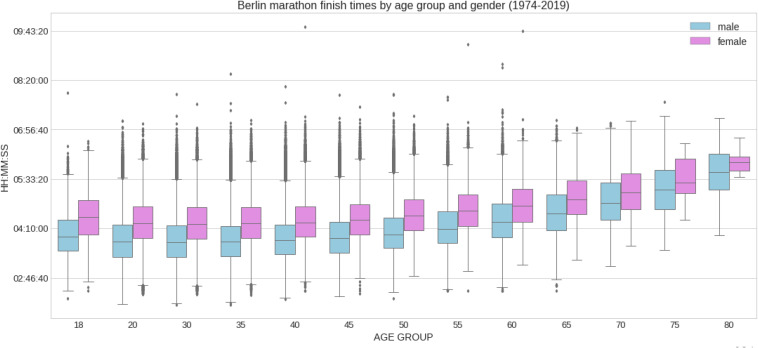
Distribution of marathon race times by age group and sex. The chart shows five key data values: minimum, first quartile (Q1), median (∼mean), third quartile (Q3), and maximum.

**FIGURE 3:**
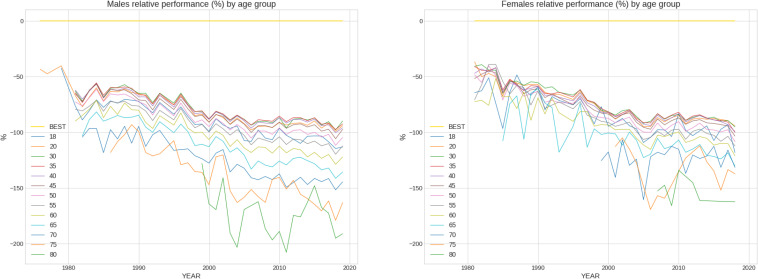
Relative male and female performance by sex and age expressed in % of the annual best time.

**FIGURE 4:**
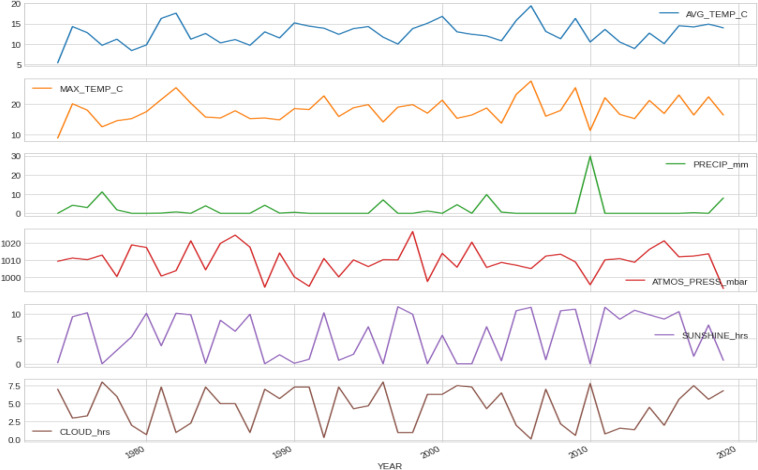
Time profiles of measured weather variables from 1974 to 2019. PRECIP, precipitation in mm; SUNSHINE duration in hours; CLOUD cover in hours; ATMOS_PRESS, atmospheric pressure in mbar; AVG_TEMP, average temperature in °Celsius; MAX_TEMP, maximum temperature in °Celsius; MN_TEMP, minimum temperature in °Celsius.

The mean finish time for the full sample was 04:25:52 ± 00:40:29 h:min:s for females and 03:56:38 ± 00:41:07 h:min:s for males. 95% confidence intervals were (04:25:40 h:min:s, 04:26:04 h:min:s) for females and (03:56:32 h:min:s, 03:56:44 h:min:s) for males (see Table 1 for a full description including mean, std, max, min and 25, 50, and 75% percentiles). Figure 1 shows the distribution of marathon race times in h:min:s for each sex. Mean race time was faster in men compared to women. Figure 2 presents the distribution of marathon race times by age group and sex. Beyond the unavoidable growing trend, the boxes tend to be bigger (taller) at both ends of the chart, appearing slightly smaller in the central age groups (40–50 years), indicating less data dispersion in the latter. Figure 3 shows the relative male and female performance by sex and age expressed in % of the annual best time. It is interesting to see how the curves for age groups 20–40 years stay consistently close together through the years, and how the age group 18 years is behind them. Figure 4 presents the time profiles of the measured weather variables between 1974 and 2019 and Table 2 shows the details of the weather variables with mean, standard deviation, minimum and maximum values.

**TABLE 1:** Basic values of the full sample for race times in h:min:s.

Full sample finish times	*n*	Mean	std	Min	25%	50%	75%	Max
Females	158,338	04:25:52	00:40:29	02:18:11	03:57:26	04:22:52	04:50:40	09:49:41
Males	711,136	03:56:38	00:41:07	02:01:39	03:28:01	03:53:00	04:21:23	08:47:19

**TABLE 2:** Details of the weather variables with mean, standard deviation, minimum, and maximum values.

	Precipitation (mm)	Sunshine (hrs.)	Cloud cover (hrs.)	Atmospheric pressure (mbar)	Average temperature (°C)	Maximum temperature (°C)
Mean	2.0	5.6	4.5	1009.8	12.7	18.0
std	5.0	4.5	2.7	8.0	2.7	3.8
Min	0.0	0.0	0.1	993.6	5.4	8.8
25%	0.0	0.7	2.0	1005.4	10.9	15.4
50%	0.0	7.0	5.0	1010.3	12.8	17.6
75%	1.1	10.0	7.0	1014.0	14.3	20.2
Max	29.8	11.4	8.0	1026.8	19.4	27.6

*Mean, average value; std, standard deviation; Min, minimum value; Max, maximum value.*

**FIGURE 5:**
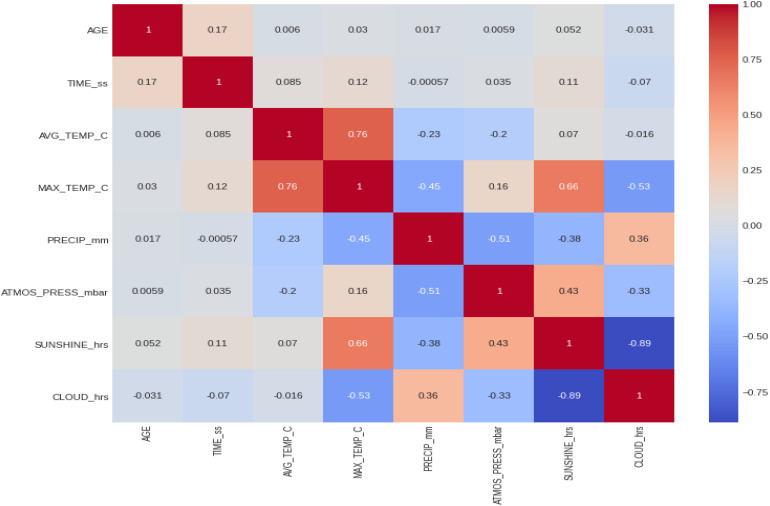
Correlation matrix of marathon race times with the descriptors (age and weather variables).

**FIGURE 6:**
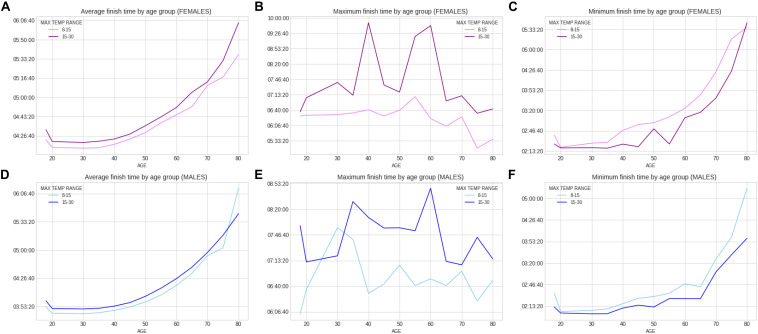
Charts of marathon race times by age group and sex for maximum temperatures on race day.

**FIGURE 7:**
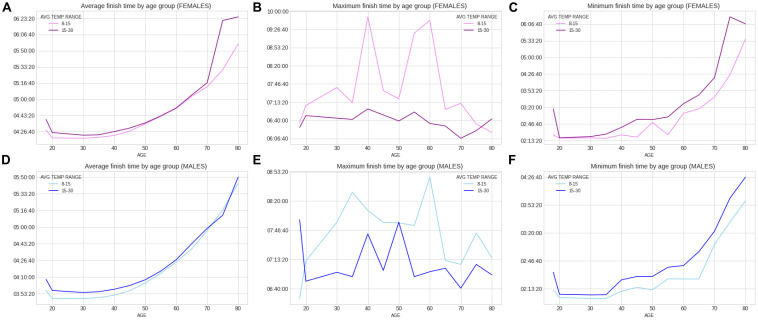
Charts of marathon race times by age group and sex for average temperatures on race day.

**FIGURE 8:**
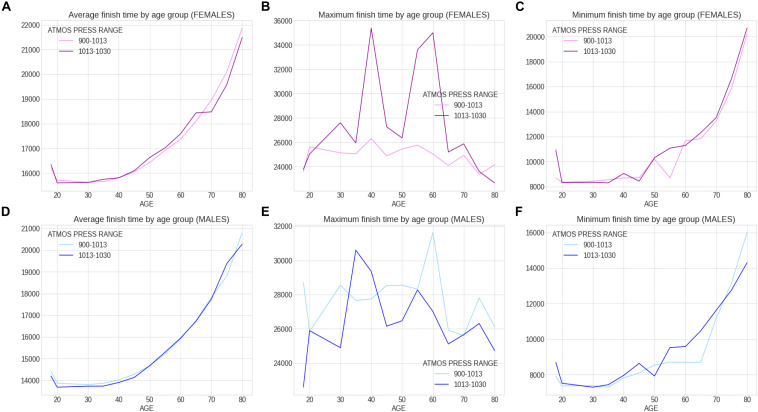
Charts of marathon race times by age group and sex for atmospheric pressure range on race day.

**FIGURE 9:**
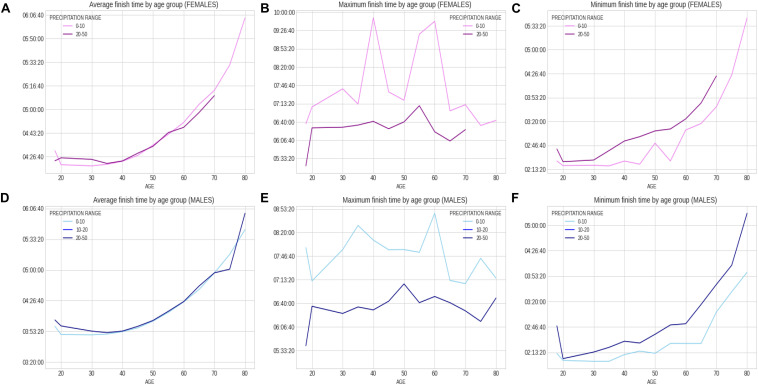
Charts of marathon race times by age group and sex for precipitation on race day.

Within the 46 years of the ‘Berlin Marathon,’ there was some minor precipitation in 18 years with 28 years without any rain; sunshine was predominant in 25 of the events, whilst in the other 21 years, cloud cover was predominant. There was no trend with time in any of the weather variables (e.g., no increase in temperature over the years). Figure 5 presents the correlation matrix of the finish times with the descriptors (age and weather variables). Marathon races times show a statistically weak (*r* = 0.17, *p* < 0.05) but positive correlation with age group, clearly shown in the earlier boxplot chart. The correlation of marathon races times with age is, however, higher for males (*r* = 0.2, *p* < 0.05) than for females (*r* = 0.148, *p* < 0.05) suggesting a larger impact of age on male performance. Other noticeable (but weaker) correlation coefficients of marathon races times are sunshine duration (*r* = 0.11, *p* < 0.05) and maximum temperature (*r* = 0.12, *p* < 0.05) where these variables were strongly correlated among them (*r* = 0.66, *p* < 0.05). Table 3 presents the analysis by maximum temperature and Figure 6 the corresponding charts of marathon race times (in seconds) by age group and sex for maximum temperatures on race day. Charts A and D (average race times) show the curves of the higher temperatures over the curves of the lower temperature indicating that marathon races times increased when temperatures increased (*p* < 0.05 for females and *p* = 0.39 for males). Charts C and F on the right side present the minimum (fastest) race times. The darker curves (higher temperatures) are below the light-colored curves for all age ranges and sex showing that those runners who achieved the fastest race times competed on race days with higher maximum temperatures (*p* < 0.05 for both males and females). This is in contrast to average race times which are more influenced by runners of all performance tiers. Also, in charts C and F (minimum race times), there seems to be a growing gap between the curves from age group 35–40 years, so the temperature difference does not seem to have an influence on younger age groups in the same way. Table 4 presents the analysis by average temperature and Figure 7 the corresponding charts of marathon race times by age group and sex for average temperatures on race day. Charts C and F show again a widening gap between the curves from age group 35 years. Table 5 presents the analysis by atmospheric pressure range and Figure 8 the charts of marathon race times by age group and sex for atmospheric pressure range on race days. Table 6 presents the analysis by precipitation and Figure 9 the charts of marathon race times by age group and sex for precipitation on race days. In the context of the negligible correlation of finish times with the precipitation, there are still some insights in the latter charts: curves on charts A and D (average finish times) stay pretty close together, so the levels of rain do not seem to affect the average finish times (*p* = 0.49 for females, *p* < 0.05 for males). Curves on C and F (minimum – or best – finish times) show a widening gap from age 20 years for males and age 30 years for females, indicating that higher precipitation levels were matched by a generalized worse performance across most age levels (*p* < 0.05 for both genders).

**TABLE 3:** Analysis by maximum temperature.

Age groups	Maximum temperature range (°C)	Marathon race time (Females)					Marathon race time (Males)				
		*n*	Mean	std	Min	Max	*n*	Mean	std	Min	Max
18	8–15	109	04:23:06	00:37:54	02:39:39	06:27:09	564	03:52:30	00:35:50	02:33:00	06:04:08
	15–30	647	04:31:59	00:41:59	02:25:00	06:36:24	4261	03:59:52	00:39:05	02:12:00	07:58:43
20	8–15	2623	04:16:44	00:39:49	02:19:41	06:28:57	10995	03:45:15	00:38:56	02:05:08	06:35:43
	15–30	21875	04:21:48	00:41:41	02:18:34	07:07:05	84717	03:50:47	00:41:29	02:02:48	07:11:36
30	8–15	2323	04:16:00	00:40:39	02:26:21	06:30:11	10403	03:44:37	00:38:04	02:06:49	07:55:55
	15–30	21306	04:20:55	00:41:13	02:18:55	07:40:08	85825	03:50:17	00:40:52	02:01:39	07:19:28
35	8–15	2674	04:16:21	00:36:38	02:27:41	06:34:03	12971	03:45:53	00:37:22	02:09:08	07:41:00
	15–30	23423	04:21:59	00:40:13	02:18:11	07:12:28	105567	03:51:02	00:39:55	02:01:41	08:30:02
40	8–15	3072	04:19:20	00:36:00	02:47:16	06:40:50	14330	03:48:32	00:36:05	02:17:10	06:30:36
	15–30	27113	04:23:57	00:39:05	02:24:54	09:49:41	116916	03:53:40	00:39:12	02:10:24	08:09:19
45	8–15	2628	04:23:48	00:35:57	02:57:00	06:27:20	11894	03:52:35	00:35:38	02:25:36	06:42:56
	15–30	23397	04:28:10	00:38:27	02:20:32	07:34:33	102378	03:57:46	00:39:18	02:15:00	07:55:39
50	8–15	1492	04:29:28	00:36:23	03:00:04	06:39:52	7719	03:58:32	00:36:05	02:28:15	07:07:24
	15–30	14860	04:35:24	00:38:46	02:49:55	07:19:21	72120	04:05:11	00:39:24	02:12:08	07:55:55
55	8–15	594	04:37:54	00:38:01	03:09:35	07:09:24	3858	04:06:38	00:37:11	02:33:23	06:40:51
	15–30	6294	04:43:02	00:40:18	02:25:00	09:19:54	36797	04:14:47	00:39:54	02:25:04	07:52:04
60	8–15	224	04:44:57	00:36:51	03:23:37	06:21:50	1854	04:18:07	00:38:32	02:48:00	06:49:34
	15–30	2498	04:51:16	00:40:27	03:08:10	09:43:23	17539	04:26:09	00:41:35	02:25:00	08:47:19
65	8–15	79	04:51:54	00:35:05	03:45:41	06:05:05	689	04:32:26	00:41:16	02:43:27	06:40:46
	15–30	825	05:04:25	00:39:54	03:17:10	07:00:00	6726	04:39:39	00:44:07	02:25:00	07:12:17
70	8–15	20	05:10:24	00:32:39	04:23:29	06:25:41	220	04:53:16	00:40:09	03:26:09	06:59:28
	15–30	202	05:13:29	00:43:09	03:40:34	07:11:15	2122	04:57:05	00:45:09	03:06:18	07:07:46
75	8–15	1	05:17:36	00:00:00	05:17:36	05:17:36	34	05:02:19	00:41:23	04:00:00	06:20:42
	15–30	48	05:31:45	00:35:52	04:24:55	06:33:20	538	05:17:26	00:46:21	03:32:53	07:43:32
80	8–15	1	05:36:59	00:00:00	05:36:59	05:36:59	4	06:13:00	00:43:57	05:15:46	06:47:37
	15–30	10	06:04:40	00:17:36	05:44:22	06:42:36	95	05:42:59	00:43:05	03:58:23	07:15:28

*Mean, average value; std, standard deviation; Min, minimum value; Max, maximum value.*

**TABLE 4:** Analysis by average temperature range.

Age groups	Average temperature range (°C)	Marathon race time (Females)					Marathon race time (Males)				
		*n*	Mean	std	Min	Max	*n*	Mean	Std	Min	Max
18	8–15	599	04:28:27	00:41:09	02:25:00	06:36:24	3800	03:56:39	00:37:53	02:12:00	06:28:50
	15–30	157	04:39:17	00:41:52	03:17:03	06:27:03	1025	04:07:44	00:40:51	02:33:00	07:58:43
20	8–15	20213	04:20:18	00:41:32	02:18:34	07:07:05	77380	03:48:38	00:41:06	02:02:48	07:11:36
	15–30	4285	04:25:47	00:41:11	02:19:12	06:48:36	18332	03:56:33	00:41:15	02:06:44	06:484
30	8–15	19538	04:19:53	00:41:31	02:18:55	07:40:08	77456	03:48:31	00:40:41	02:01:39	07:55:55
	15–30	4091	04:23:03	00:39:28	02:21:34	06:44:00	18772	03:54:29	00:39:59	02:05:56	06:58:45
35	8–15	21236	04:20:56	00:40:28	02:18:11	07:12:28	94191	03:49:14	00:39:45	02:01:41	08:30:02
	15–30	4861	04:23:24	00:37:17	02:26:24	06:41:48	24347	03:55:18	00:39:04	02:06:08	06:53:43
40	8–15	24475	04:22:43	00:39:07	02:24:54	09:49:41	104255	03:51:54	00:38:54	02:10:24	08:09:19
	15–30	5710	04:26:46	00:37:18	02:40:05	07:00:54	26991	03:57:45	00:38:33	02:23:56	07:42:28
45	8–15	21682	04:27:12	00:38:33	02:20:32	07:34:33	92967	03:56:15	00:39:02	02:15:00	07:55:39
	15–30	4343	04:30:24	00:36:28	02:56:00	06:49:53	21305	04:01:31	00:38:24	02:28:00	07:00:52
50	8–15	13973	04:34:43	00:38:53	02:49:55	07:19:21	65807	04:03:59	00:39:16	02:12:08	07:55:06
	15–30	2379	04:35:43	00:36:51	02:55:37	06:38:54	14032	04:07:11	00:38:29	02:28:00	07:55:55
55	8–15	5974	04:42:33	00:40:30	02:25:00	09:19:54	33928	04:13:39	00:39:54	02:25:04	07:52:04
	15–30	914	04:42:51	00:37:37	03:00:52	06:55:14	6727	04:15:51	00:38:44	02:39:00	06:53:43
60	8–15	2377	04:50:42	00:40:34	03:08:10	09:43:23	16081	04:25:00	00:41:27	02:25:00	08:47:19
	15–30	345	04:51:02	00:37:37	03:27:22	06:34:23	3312	04:27:16	00:40:58	02:41:00	06:59:23
65	8–15	762	05:03:07	00:40:29	03:17:10	07:00:00	6090	04:38:03	00:44:01	02:25:00	07:12:17
	15–30	142	05:04:23	00:34:59	03:45:00	06:29:41	1325	04:43:15	00:43:09	02:57:45	07:03:21
70	8–15	203	05:12:51	00:43:09	03:40:34	07:11:15	2021	04:56:25	00:44:55	03:06:18	07:07:46
	15–30	19	05:17:08	00:31:54	04:19:23	06:07:10	321	04:58:40	00:43:20	03:21:53	06:40:36
75	8–15	48	05:30:25	00:35:10	04:24:55	06:33:20	515	05:17:04	00:46:07	03:32:53	07:43:32
	15–30	1	06:21:10	00:00:00	06:21:10	06:21:10	57	05:11:38	00:46:50	04:01:33	07:07:44
80	8–15	9	05:57:05	00:14:07	05:36:59	06:17:46	88	05:43:29	00:42:16	03:58:23	07:15:28
	15–30	2	06:24:54	00:25:01	06:07:13	06:42:36	11	05:49:50	00:52:47	04:26:42	06:55:52

*Mean, average value; std, standard deviation; Min, minimum value; Max, maximum value.*

**TABLE 5:** Analysis by atmospheric pressure range.

Age group	Atmospheric pressure range (mbar)	Marathon race time (Females)					Marathon race time (Males)				
		*n*	Mean	std	Min	Max	*n*	Mean	Std	Min	Max
18	900–1013	524	04:29:56	00:42:28	02:25:00	06:33:14	3101	04:00:19	00:39:02	02:12:00	07:58:43
	1013–1030	232	04:32:25	00:39:18	03:02:00	06:36:24	1724	03:56:39	00:38:15	02:25:00	06:16:30
20	900–1013	16563	04:21:51	00:40:54	02:19:12	07:07:05	64548	03:51:09	00:41:07	02:02:48	07:10:51
	1013–1030	7935	04:20:02	00:42:45	02:18:34	06:57:20	31164	03:48:03	00:41:25	02:05:21	07:11:36
30	900–1013	15959	04:20:30	00:40:46	02:20:23	06:58:53	67079	03:50:02	00:40:17	02:03:03	07:55:55
	1013–1030	7670	04:20:17	00:42:03	02:18:55	07:40:08	29149	03:48:51	00:41:20	02:01:39	06:55:02
35	900–1013	17822	04:20:54	00:38:49	02:22:18	06:57:37	83401	03:51:08	00:39:18	02:01:41	07:41:00
	1013–1030	8275	04:22:28	00:42:07	02:18:11	07:12:28	35137	03:48:54	00:40:33	02:03:59	08:30:02
40	900–1013	20874	04:23:29	00:38:03	02:24:54	07:18:23	93566	03:53:40	00:38:40	02:10:24	07:42:28
	1013–1030	9311	04:23:29	00:40:27	02:30:47	09:49:41	37680	03:51:43	00:39:27	02:12:45	08:09:19
45	900–1013	17898	04:27:29	00:37:09	02:25:00	06:54:45	80309	03:57:57	00:38:37	02:15:00	07:55:39
	1013–1030	8127	04:28:17	00:40:31	02:20:32	07:34:33	33963	03:55:33	00:39:46	02:24:00	07:15:57
50	900–1013	11134	04:33:46	00:37:37	02:49:55	07:04:09	56512	04:04:31	00:38:40	02:22:23	07:55:55
	1013–1030	5218	04:37:11	00:40:30	02:51:47	07:19:21	23327	04:04:35	00:40:17	02:12:08	07:21:15
55	900–1013	4545	04:42:01	00:39:11	02:25:00	07:09:24	28746	04:13:36	00:39:15	02:25:04	07:52:04
	1013–1030	2343	04:43:41	00:41:54	03:04:31	09:19:54	11909	04:15:00	00:40:48	02:38:52	07:51:18
60	900–1013	1793	04:49:30	00:38:49	03:14:10	06:57:25	13498	04:25:14	00:41:10	02:25:00	08:47:19
	1013–1030	929	04:53:08	00:42:40	03:08:10	09:43:23	5895	04:25:43	00:41:50	02:39:42	07:30:15
65	900–1013	624	05:01:33	00:38:27	03:17:10	06:41:28	5301	04:39:06	00:43:41	02:25:00	07:12:17
	1013–1030	280	05:07:15	00:42:01	03:25:12	07:00:00	2114	04:38:40	00:44:27	02:54:35	06:58:40
70	900–1013	149	05:15:47	00:42:42	03:40:34	06:55:36	1651	04:57:08	00:44:17	03:06:18	07:06:50
	1013–1030	73	05:07:57	00:41:09	03:45:14	07:11:15	691	04:55:43	00:45:43	03:13:45	07:07:46
75	900–1013	29	05:34:58	00:37:27	04:24:55	06:29:33	381	05:13:17	00:46:44	03:39:39	07:43:32
	1013–1030	20	05:26:21	00:32:52	04:37:51	06:33:20	191	05:23:00	00:44:28	03:32:53	07:18:33
80	900–1013	7	06:04:26	00:21:19	05:36:59	06:42:36	69	05:46:55	00:44:10	04:26:42	07:15:28
	1013–1030	4	05:58:09	00:14:49	05:44:47	06:17:46	30	05:37:56	00:41:16	03:58:23	06:52:16

*Mean, average value; std, standard deviation; Min, minimum value; Max, maximum value.*

**TABLE 6:** Analysis by precipitation.

Age group	Precipitation range (mm)	Marathon race time (Females)					Marathon race time (Males)				
		*n*	Mean	std	Min	Max	*n*	Mean	Std	Min	Max
18	0–10	723	04:31:01	00:41:51	02:25:00	06:36:24	4727	03:58:52	00:38:51	02:12:00	07:58:43
	20–50	33	04:23:49	00:33:06	02:41:41	05:19:39	98	04:05:44	00:35:08	02:48:00	05:39:52
20	0–10	23475	04:21:04	00:41:35	02:18:34	07:07:05	92975	03:49:56	00:41:14	02:02:48	07:11:36
	10–20	–	–	–	–	–	126	03:20:52	00:32:05	02:16:20	05:17:56
	20–50	1023	04:25:54	00:39:36	02:23:58	06:28:57	2611	03:59:15	00:40:38	02:05:08	06:35:43
30	0–10	22633	04:20:14	00:41:12	02:18:55	07:40:08	93181	03:49:33	00:40:40	02:01:39	07:55:55
	20–50	996	04:24:50	00:40:29	02:26:21	06:30:11	3047	03:53:37	00:38:44	02:13:46	06:25:35
35	0–10	24996	04:21:23	00:40:00	02:18:11	07:12:28	114432	03:50:25	00:39:46	02:01:41	08:30:02
	20–50	1101	04:21:56	00:37:31	02:39:29	06:34:03	4106	03:51:58	00:37:32	02:19:55	06:34:45
40	0–10	28741	04:23:28	00:38:54	02:24:54	09:49:41	125719	03:53:05	00:39:01	02:10:24	08:09:19
	20–50	1444	04:23:43	00:36:44	02:52:49	06:40:50	5527	03:53:38	00:36:13	02:27:58	06:30:36
45	0–10	24635	04:27:39	00:38:18	02:20:32	07:34:33	109305	03:57:10	00:39:07	02:15:00	07:55:39
	20–50	1390	04:29:09	00:36:56	02:58:51	06:27:20	4967	03:58:47	00:35:39	02:25:36	06:42:56
50	0–10	15485	04:34:54	00:38:43	02:49:55	07:19:21	76519	04:04:31	00:39:15	02:12:08	07:55:55
	20–50	867	04:34:04	00:36:32	03:06:53	06:39:52	3320	04:05:13	00:36:42	02:37:03	07:07:24
55	0–10	6528	04:42:32	00:40:11	02:25:00	09:19:54	39042	04:13:58	00:39:49	02:25:04	07:52:04
	20–50	360	04:43:44	00:39:19	03:09:35	07:09:24	1613	04:15:12	00:37:09	02:49:17	06:40:51
60	0–10	2602	04:50:54	00:40:20	03:08:10	09:43:23	18589	04:25:22	00:41:32	02:25:00	08:47:19
	20–50	120	04:47:24	00:37:08	03:23:37	06:21:50	804	04:25:42	00:37:33	02:50:53	06:49:34
65	0–10	858	05:03:37	00:39:54	03:17:10	07:00:00	7100	04:38:49	00:44:04	02:25:00	07:12:17
	20–50	46	04:57:51	00:34:39	03:45:41	06:05:05	315	04:42:32	00:39:45	03:16:07	06:40:46
70	0–10	206	05:13:29	00:42:53	03:40:34	07:11:15	2191	04:56:43	00:45:06	03:06:18	07:07:46
	20–50	16	05:09:46	00:34:14	04:23:29	06:25:41	151	04:56:53	00:38:36	03:42:27	06:29:17
75	0–10	49	05:31:27	00:35:33	04:24:55	06:33:20	554	05:17:02	00:46:22	03:32:53	07:43:32
	20–50	–	–	–	–	–	18	05:00:53	00:37:26	04:07:38	06:14:19
80	0–10	10	06:04:40	00:17:36	05:44:22	06:42:36	97	05:43:50	00:43:10	03:58:23	07:15:28
	20–50	1	05:36:59	00:00:00	05:36:59	05:36:59	2	06:01:36	01:04:49	05:15:46	06:47:26

*Mean, average value; std, standard deviation; min, minimum value; max, maximum value.*

## Discussion

This study investigated the influence of environmental conditions such as temperature, sunshine duration, precipitation, barometric pressure on marathon race times in age group marathoners with the assumption that higher ambient temperatures would impair marathon performance in athletes in master marathoners.

The main findings were (*i*) marathon race times were significantly and positively related with age where the correlation was higher for males than for females, (*ii*) marathon race times were significantly and positively correlated with both sunshine duration and daily maximum temperature with no differences between the sexes, (*iii*) the fastest marathon runners achieved the fastest race times on race days with higher maximum temperatures with no differences between the sexes, (*iv*) increased daily maximum temperatures decreased race performance in age group marathoners from age group 35–40 years and older with no differences between the sexes, and (*v*) higher precipitation levels impaired top performance across most age groups with no differences between the sexes.

We found different influences of temperature on different performance levels (i.e., younger and age group runners) with no differences between the sexes. Marathon race times of the whole field of runners were significantly and positively related with both sunshine duration and daily maximum temperature, where sunshine duration and daily maximum temperature were highly correlated. The fastest marathon runners, however, achieved the fastest race times on race days with higher maximum temperatures. In contrast, regarding age group marathoners, the daily maximum temperatures showed an influence on age group marathoners from age group 35–40 years and older with no differences between the sexes.

It is well-known that marathon race times are impaired with increasing temperatures (Trapasso and Cooper, 1989; Cheuvront and Haymes, 2001; Ely et al., 2007b; González-Alonso, 2007; Maughan, 2010; El Helou et al., 2012; Nikolaidis et al., 2019). The temperature on race day has, however, a different influence on slower and faster runners (Montain et al., 2007; Ely et al., 2008; El Helou et al., 2012) where the optimum temperature for a fast marathon race time may be lower for faster runners than for slower runners (Maughan, 2010).

Since elite marathoners are generally faster than master marathoners (Lepers and Cattagni, 2012), higher ambient temperatures might have a higher impact on master marathoners compared to elite marathoners. However, higher temperatures seemed to slow down faster runners compared to slower runners. A study investigating the influence of air temperature on female marathoners of different performance levels showed that increasing air temperatures slowed pace more in faster runners (winner, 25th place) than slower runners (50th place, 100th place) (Ely et al., 2008). The disparate findings compared to our findings that the fastest marathon runners achieved the fastest race times on race days with higher maximum temperatures might be explained by the different statistical approach and the fact that Ely et al. (2008) investigated only female marathoners, whereas we investigated both female and male marathoners. Furthermore, Ely et al. (2008) analyzed national championship marathon runners which were all very fast comparing to our age group runners. And their slower runners were actually quite fast comparing to the recreational runners (Ely et al., 2008). We investigated both female and male marathoners with no differences between the sexes. Future studies might investigate other large city marathons regarding this aspect.

In contrast, it has also been shown that slower runners suffered a greater performance decline in higher temperatures than faster runners. An analysis regarding the effects of air temperature on performance in marathoners competing in the ‘Stockholm Marathon’ between 1980 and 2008 showed that slower runners were more affected by unfavorable weather conditions than faster runners (Vihma, 2010). Air temperature was the single weather parameter with the highest correlation with finishing time anomaly (i.e., deviation of the annual finishing time from the linear trend of the finishing time) with the highest values for slowest runners (Vihma, 2010). Regarding the sexes, the effects of warm weather were less evident for female than for male runners (Vihma, 2010). The finding of Vihma (Vihma, 2010) is in line with our finding that race times of the whole field of runners (i.e., the general mass) was related with both sunshine duration and daily maximum temperature (i.e., daily maximum temperature increased with increasing sunshine duration).

Apart the influence from temperature, we also found an influence of precipitations on marathon race times. Higher precipitation levels impaired performance across most older age groups (i.e., master marathoners older than 35–40 years and older) with no differences between the sexes. The influence of precipitation has been investigated in the ‘Stockholm Marathon’ (Vihma, 2010) and in the ‘Boston Marathon’ (Knechtle et al., 2019; Nikolaidis et al., 2019) for marathoners of different performance levels, but not for age group marathoners. Vihma investigated the effects of different variables (i.e., air temperature, relative and specific humidity, wind speed, solar shortwave radiation, thermal longwave radiation, and rain) on the performance of female and male participants in the annual ‘Stockholm Marathon’ from 1980 to 2008 (Vihma, 2010). The occurrence of rain was related to finishing time anomaly, expressed as deviation of the annual finishing time from the linear trend of the finishing time. However, the effects of rain only arose from the negative correlation the air temperature Vihma (Vihma, 2010). A study examining the relationship of weather conditions with running performance in the Boston Marathon from 1972 to 2018 showed that increasing precipitation was significantly related to slower performances in runners of all performance levels, except the annual winners (Knechtle et al., 2019). Also, when male performances in the ‘Boston Marathon’ from 1897 to 2018 were investigated, increasing precipitations worsened performances of both the top 100 and the top 10 finishers (Nikolaidis et al., 2019). Obviously, precipitation has only an influence on slower runners, but not on faster runners, regarding our findings from the ‘Berlin Marathon’ and the findings from ‘Boston Marathon’ (Knechtle et al., 2019).

The impact of precipitation on marathon performance might be attributed to the role of thermoregulation and particularly, to the association of higher humidity with reduced capacity to lose heat using evaporation (Wilmore and Costill, 1999). Humidity has been suggested as a factor of heat dissipation, and consequently, an increase of humidity would affect negatively the balance between heat production ad dissipation (Bouscaren et al., 2019). In addition, the thermoregulation has been well-documented to be impaired with aging reducing sport performance for master athletes especially in hot environmental conditions (Kenney et al., 2020). These age-related differences in thermoregulation might be attributed to differences in performance related characteristics (e.g., physical fitness and body composition) (Kenney and Munce, 2003).

We also found that marathon race times of the whole field of runners were significantly and positively related with age where the correlation was higher for male than for female marathoners suggesting a higher impact of age on male than female performance. An effect of age on marathon performance has already been reported (Nikolaidis and Knechtle, 2017; Stones, 2019). Stones investigated in his analysis the top 100 age group performances in master marathoners and found higher performance times for women than for men where the performance decline was greater at older ages and in women than men (Stones, 2019). The disparate findings that we found a higher impact on male performance with age and Stones a greater decline in performance at higher ages in women might be explained by the different samples that were investigated (i.e., the whole field of marathon race over decades compared to the top 100 age group performances). When the performance was expressed in percent of the annual fastest, we found that performance of female and male athletes in age groups 20–40 years remained very close over years and an increasing drop in performance with increasing age over calendar years. The gradual drop in relative performance through the years is most likely explained by the increasingly popular character of the marathon where every year more and more recreational runners join it. This is very obvious in the very old age groups (i.e., 75 years and older).

### Limitations

Since the first edition of ‘Berlin Marathon’, several changes occurred such as moving the race course from outside Berlin into the city of Berlin, changing the start procedure from mass start in the beginning to start in blocks by performance levels, moving race start from afternoon to morning, aid stations with food and drinks, and including professional runners. Most of these changes cannot be included in this kind of performance analysis. Regarding temperature, historic weather data had only average, minimum (morning) and maximum (afternoon) values for a day. There might also have been a change of measurement in weather data during the decades, which also may have an impact on the results. Future studies might investigate the influence of temperature considering temperatures at hourly intervals during race time. A further limitation is the introduction of electronic time measurement in recent years. Before this area, slower runners had to wait and walk for the start line until the large mass of runners has started. This difference between ‘gun time’ and ‘chip time’ leads to partially massive differences especially in the race time of slower and older runners. Race data from 1978 and 1980 are missing in the race results from ‘Berlin Marathon’ which could also have an influence on our analysis.

## Conclusion

In summary, higher daily maximum temperatures and higher precipitation levels impaired performance of master marathoners (35–40 years and older) competing in the ‘Berlin Marathon’ in the last 45 years. For master marathoners, temperatures below 15 °C and no precipitation would be beneficial for a fast marathon race time. The findings of an impaired performance in older age groups might also be due to the continuous decrease in performance in older age groups across calendar years.

## Data Availability Statement

The raw data supporting the conclusions of this article will be made available by the authors, without undue reservation.

## Author Contributions

BK and VS drafted the manuscript. DV performed the statistical analyses. EV collected the all data. JA-C, PN, IC, and TR helped in drafting the final version of the manuscript. All the authors contributed to the article and approved the submitted version.

## Conflict of Interest

The authors declare that the research was conducted in the absence of any commercial or financial relationships that could be construed as a potential conflict of interest.

## Footnotes

1 www.runnersworld.com/races-places/a20823734/these-are-the-worlds-fastest-marathoners-and-marathon-courses/

2 www.bmw-berlin-marathon.com/impressionen/statistik-und-geschichte/ergebnisarchiv/

3 www.dwd.de/DE/leistungen/klimadatendeutschland/klarchivtagmonat.html?nn=16102

4 https://colab.research.google.com/

5 www.python.org

## References

[B1] Ahmadyar B. Rosemann T. Rüst C. A. Knechtle B. (2016). Improved race times in marathoners older than 75 years in the last 25 years in the world’s largest marathons. *Chin. J. Physiol.* 59 139–147. 10.4077/cjp.2016.bae382 27188466

[B2] Ahmadyar B. Rüst C. A. Rosemann T. Knechtle B. (2015). Participation and performance trends in elderly marathoners in four of the world’s largest marathons during 2004-2011. *Springerplus* 4:465. 10.1186/s40064-015-1254-6 26339566 PMC4552708

[B3] Balmain B. N. Sabapathy S. Louis M. Morris N. R. (2018). Aging and thermoregulatory control: the clinical implications of exercising under heat stress in older individuals. *Biomed Res. Int.* 2018:8306154. 10.1155/2018/8306154 30155483 PMC6098859

[B4] Bongers C. C. Eijsvogels T. M. Nyakayiru J. Veltmeijer M. T. Thijssen D. H. Hopman M. T. (2014). Thermoregulation and fluid balance during a 30-km march in 60- versus 80-year-old subjects. *Age (Dordr.)* 36:9725. 10.1007/s11357-014-9725-1 25403156 PMC4234746

[B5] Bouscaren N. Millet G. Y. Racinais S. (2019). Heat stress challenges in marathon vs. ultra-endurance running. *Front. Sports Act. Living* 1:59. 10.3389/fspor.2019.00059 33344982 PMC7739648

[B6] Cheuvront S. N. Haymes E. M. (2001). Thermoregulation and marathon running biological and environmental influences. *Sports Med.* 31 743–762. 10.2165/00007256-200131100-00004 11547895

[B7] El Helou N. Tafflet M. Berthelot G. Tolaini J. Marc A. Guillaume M. (2012). Impact of environmental parameters on marathon running performance. *PLoS One* 7:e37407. 10.1371/journal.pone.0037407 22649525 PMC3359364

[B8] Ely M. R. Cheuvront S. N. Montain S. J. (2007a). Neither cloud cover nor low solar loads are associated with fast marathon performance. *Med. Sci. Sports Exerc.* 39 2029–2035. 10.1249/mss.0b013e318149f2c3 17986912

[B9] Ely M. R. Cheuvront S. N. Roberts W. O. Montain S. J. (2007b). Impact of weather on marathon-running performance. *Med. Sci. Sports Exerc.* 39 487–493. 10.1249/mss.0b013e31802d3aba 17473775

[B10] Ely M. R. Martin D. E. Cheuvront S. N. Montain S. J. (2008). Effect of ambient temperature on marathon pacing is dependent on runner ability. *Med. Sci. Sports Exerc.* 40 1675–1680. 10.1249/MSS.0b013e3181788da9 18685522

[B11] González-Alonso J. (2007). Hyperthermia impairs brain, heart and muscle function in exercising humans. *Sports Med.* 37 371–373. 10.2165/00007256-200737040-00025 17465611

[B12] Jokl P. Sethi P. M. Cooper A. J. (2004). Master’s performance in the New York City Marathon 1983-1999. *Br. J. Sports Med.* 38 408–412. 10.1136/bjsm.2002.003566 15273172 PMC1724857

[B13] Kenney W. L. Munce T. A. (2003). Invited review: aging and human temperature regulation. *J. Appl. Physiol. (1985)* 95 2598–2603. 10.1152/japplphysiol.00202.2003 14600165

[B14] Kenney W. L. Wolf S. T. Dillon G. A. Berry C. W. Alexander L. M. (2020). Temperature regulation during exercise in the heat: insights for the aging athlete. *J. Sci. Med. Sport.* 10.1016/j.jsams.2020.12.007 [Epub ahead of print]. 33358656 PMC8222413

[B15] Knechtle B. Gangi S. D. Rust C. A. Villiger E. Rosemann T. Nikolaidis P. T. (2019). The role of weather conditions on running performance in the Boston Marathon from 1972 to 2018. *PLoS One* 14:e0212797. 10.1371/journal.pone.0212797 30849085 PMC6407773

[B16] Larose J. Boulay P. Sigal R. J. Wright H. E. Kenny G. P. (2013). Age-related decrements in heat dissipation during physical activity occur as early as the age of 40. *PLoS One* 8:e83148. 10.1371/journal.pone.0083148 24349447 PMC3861480

[B17] Lepers R. Cattagni T. (2012). Do older athletes reach limits in their performance during marathon running? *Age (Dordr.)* 34 773–781. 10.1007/s11357-011-9271-z 21617894 PMC3337940

[B18] Maffetone P. B. Malcata R. Rivera I. Laursen P. B. (2017). The Boston Marathon versus the world marathon majors. *PLoS One* 12:e0184024. 10.1371/journal.pone.0184024 28863152 PMC5581174

[B19] Maughan R. J. (2010). Distance running in hot environments: a thermal challenge to the elite runner. *Scand. J. Med. Sci. Sports* 20(Suppl. 3) 95–102. 10.1111/j.1600-0838.2010.01214.x 21029196

[B20] McGinn R. Poirier M. P. Louie J. C. Sigal R. J. Boulay P. Flouris A. D. (2017). Increasing age is a major risk factor for susceptibility to heat stress during physical activity. *Appl. Physiol. Nutr. Metab.* 42 1232–1235. 10.1139/apnm-2017-0322 28750177

[B21] Montain S. J. Ely M. R. Cheuvront S. N. (2007). Marathon performance in thermally stressing conditions. *Sports Med.* 37 320–323. 10.2165/00007256-200737040-00012 17465598

[B22] Nikolaidis P. T. di Gangi S. Chtourou H. Rüst C. A. Rosemann T. Knechtle B. (2019). The role of environmental conditions on marathon running performance in men competing in Boston Marathon from 1897 to 2018. *Int. J. Environ. Res. Public Health* 16:614. 10.3390/ijerph16040614 30791523 PMC6406844

[B23] Nikolaidis P. T. Knechtle B. (2017). Effect of age and performance on pacing of marathon runners. *Open Access J. Sports Med.* 8 171–180. 10.2147/oajsm.s141649 28860876 PMC5571841

[B24] Stapleton J. M. Poirier M. P. Flouris A. D. Boulay P. Sigal R. J. Malcolm J. (2015). Aging impairs heat loss, but when does it matter? *J. Appl. Physiol. (1985)* 118 299–309. 10.1152/japplphysiol.00722.2014 25505030 PMC4312844

[B25] Stones M. J. (2019). Age differences, age changes, and generalizability in marathon running by master athletes. *Front. Psychol.* 10:2161. 10.3389/fpsyg.2019.02161 31616350 PMC6764238

[B26] Trapasso L. M. Cooper J. D. (1989). Record performances at the Boston Marathon: biometeorological factors. *Int. J. Biometeorol.* 33 233–237. 10.1007/bf01051083 2613367

[B27] Vihma T. (2010). Effects of weather on the performance of marathon runners. *Int. J. Biometeorol.* 54 297–306. 10.1007/s00484-009-0280-x 19937453

[B28] Vitti A. Nikolaidis P. T. Villiger E. Onywera V. Knechtle B. (2020). The “New York City Marathon”: participation and performance trends of 1.2M runners during half-century. *Res. Sports Med.* 28 121–137. 10.1080/15438627.2019.1586705 30889965

[B29] Wilmore J. H. Costill D. L. (1999). *Physiology of Sport and Exercise.* Champaign, IL: Human Kinetics.

[B30] Zhang S. Meng G. Wang Y. Li J. (1992). Study of the relationships between weather conditions and the marathon race, and of meteorotropic effects on distance runners. *Int. J. Biometeorol.* 36 63–68. 10.1007/bf01208915 1634282

